# Defecography by digital radiography: experience in clinical practice*

**DOI:** 10.1590/0100-3984.2015.0108

**Published:** 2016

**Authors:** Amanda Nogueira de Sá Gonçalves, Marco Aurélio Sousa Sala, Rodrigo Ciotola Bruno, José Alberto Cunha Xavier, João Mauricio Canavezi Indiani, Marcelo Fontalvo Martin, Paulo Maurício Chagas Bruno, Marcelo Souto Nacif

**Affiliations:** 1 Physician at the Unidade de Radiologia Clínica (URC) and at the Hospital Vivalle/D'Or/São Luiz, São José dos Campos, SP, Brazil.; 2 General Surgeon and Coloproctologist at the Hospital Vivalle/D'Or/São Luiz, São José dos Campos, SP, Brazil.; 3 General Surgeon and Coloproctologist at the Hospital Pio XII, São José dos Campos, SP, Brazil.; 4 Adjunct Professor in the Radiology Department of the Faculdade de Medicina da Universidade Federal Fluminense (UFF), Niterói, RJ, Brazil.

**Keywords:** Defecography, Constipation, Rectocele, Rectal prolapse

## Abstract

**Objective:**

The objective of this study was to profile patients who undergo defecography, by age and gender, as well as to describe the main imaging and diagnostic findings in this population.

**Materials and Methods:**

This was a retrospective, descriptive study of 39 patients, conducted between January 2012 and February 2014. The patients were evaluated in terms of age, gender, and diagnosis. They were stratified by age, and continuous variables are expressed as mean ± standard deviation. All possible quantitative defecography variables were evaluated, including rectal evacuation, perineal descent, and measures of the anal canal.

**Results:**

The majority (95%) of the patients were female. Patient ages ranged from 18 to 82 years (mean age, 52 ± 13 years): 10 patients were under 40 years of age; 18 were between 40 and 60 years of age; and 11 were over 60 years of age. All 39 of the patients evaluated had abnormal radiological findings. The most prevalent diagnoses were rectocele (in 77%) and enterocele (in 38%). Less prevalent diagnoses were vaginal prolapse, uterine prolapse, and Meckel's diverticulum (in 2%, for all).

**Conclusion:**

Although defecography is performed more often in women, both genders can benefit from the test. Defecography can be performed in order to detect complex disorders such as uterine and rectal prolapse, as well as to detect basic clinical conditions such as rectocele or enterocele.

## Abstract (pt)

**Objetivo:**

Este estudo tem por objetivo descrever o perfil da população que se submeteu a exame de defecografia, de acordo com a faixa etária e sexo do paciente, bem como os principais achados e diagnósticos de imagem nesta população.

**Materiais e Métodos:**

Estudo retrospectivo e descritivo com 39 pacientes realizado entre janeiro de 2012 e fevereiro de 2014. Os pacientes foram avaliados quanto a idade, sexo e os diagnósticos encontrados. Foram divididos por idade, e as variáveis contínuas são expressas como média ± desvio-padrão. Todas as quantificações possíveis em defecografia foram realizadas neste trabalho, incluindo esvaziamento da ampola retal, descenso perineal e as medidas do canal anal.

**Resultados:**

Dos 39 pacientes estudados, todos apresentaram alterações radiológicas, sendo a maioria do sexo feminino (95%). O mais novo tinha 18 anos e o mais velho, 82 anos (idade média de 52 ± 13 anos). Dez pacientes tinham menos de 40 anos, 18 tinham entre 40 e 60 anos e 11 pacientes tinham mais do que 60 anos. Os diagnósticos mais prevalentes foram retocele anterior (77%) e enterocele (38%). Os menos frequentes foram prolapso vaginal (2%), prolapso uterino (2%) e divertículo de Meckel (2%).

**Conclusão:**

As mulheres realizam mais exames de defecografia, porém ambos os gêneros se beneficiam deste estudo. A defecografia é capaz de detectar causas complexas de distúrbio da defecação como prolapso vaginal, uterino e retal, e pode ser utilizada em condições clínicas mais simples como a avaliação de retocele ou enterocele.

## INTRODUCTION

Defecography is a radiographic method for the study of defecation that provides images of morphological and functional changes in the pelvis and anorectal segment. It is a valuable method for the study of the physiology of the pelvic dynamics of colorectal disorders such as dyskinesia, constipation, fecal incontinence, anal pain, and tenesmus^(1)^.

The first reports of radiological studies of the pelvic dynamics during evacuation were by Lennart Walldén, in 1952. However, it was only after the studies conducted by Mathieu et al., in 1984, that the test sparked interest within the medical community^(2)^.

Anorectal disorders represent a common clinical problem and have a great impact on the quality of life of the patients^(3)^. The physical examination is often difficult and provides few details, failing to identify pelvic organ prolapse in 45-90% of cases. In addition, the physical examination can fail to diagnose associated prolapses. Multiple compartment dysfunction is common and changes the surgical approach; if undiagnosed, such dysfunction leads to symptom recurrence^(1)^.

Although there is a considerable variation among treatment facilities in relation to the examination technique employed, most employ a technique based on the method standardized by Mahieu et al.^(2)^.

There have been few studies focusing on defecography tests that involve the use of conventional radiography. Therefore, the aim of the present study was to describe the profile of a population undergoing defecography, as well as the main findings of and diagnoses made from imaging studies in this population.

## MATERIALS AND METHODS

### Selection of patients

This was a descriptive, retrospective study, using data collected between January 2012 and February 2014. A total of 39 defecography tests by digital radiography, performed at the Unidade de Radiologia Clínica (URC), in the city of São José dos Campos, SP, Brazil, were selected for analysis.

### Protocol for defecography by conventional radiography

In the initial evaluation, a complete patient history should be taken in order to investigate previous intestinal diseases, pelvic or abdominal surgery, parity, and clinical condition. The procedure must be clearly explained to the patient, with the intention to obtain full cooperation and an ideal end result.

Approximately 2 hours before the exam, the patient receives 400 mL of pulverized oral contrast, in order to visualize the entire small intestine. The patient is then placed in the left lateral decubitus position for rectal administration of a barium paste to a volume of approximately 200 mL or until the patient reports discomfort (sensation of rectal fullness). The pulverized contrast should be standardized as to its density and viscosity^(1)^.

**Figure 1:**
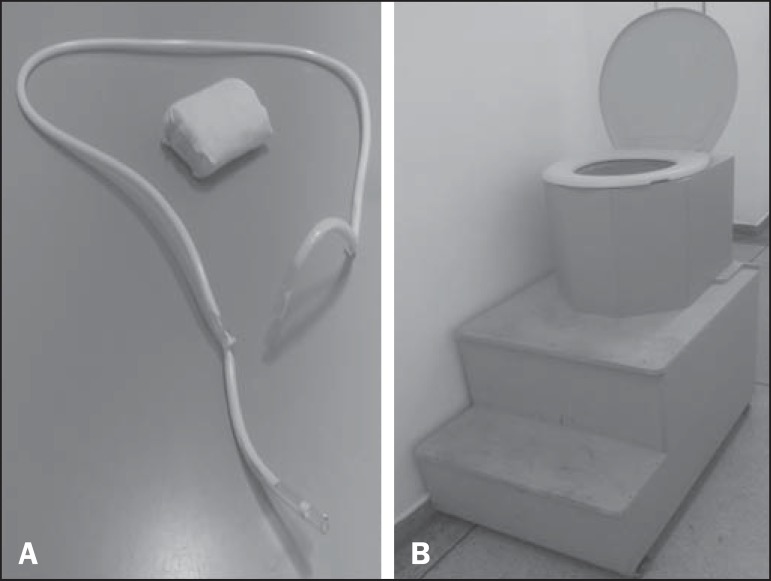
Pubococcygeus marker used for reference during the exam (**A**) and the adaptation of equipment to perform the defecography (**B**) used at our facility.

A 2 cm-high rectangular marker is placed over the pubis, in order to facilitate the identification of bone repair and serve as a reference for the quantitative analysis. Another, tubular marker (saline applicator), approximately 0.5 cm in diameter and filled with barium, is adhered to the first, running from the pubis to the sacrum and secured at both ends with hypoallergenic tape (Figure 1A). It is important that this marker is well adhered to the skin and the perianal region, so that the position of the anal canal is well recognized. The patient then sits on a radiolucent seat and remains in that position throughout the test. A wooden table that provides good exposure of the rectum is used for support (Figure 1B).

**Figure 2:**
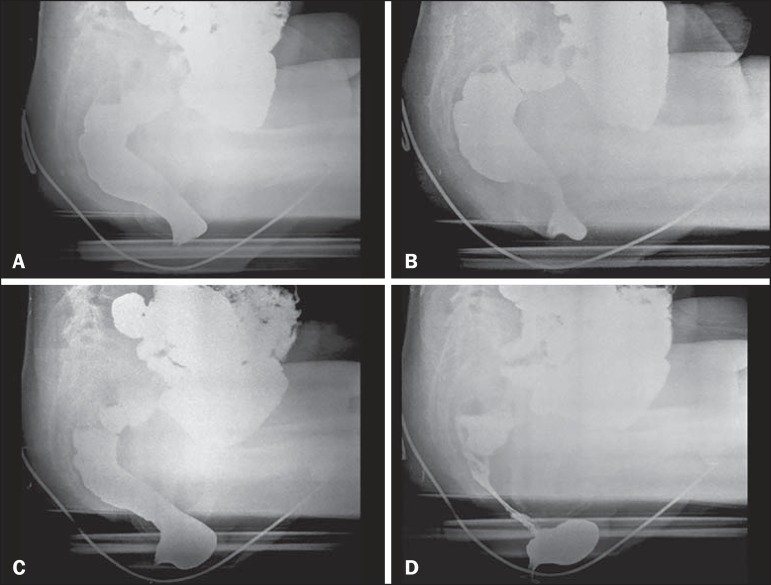
Maneuvers during the defecography examination: at rest (**A**), during contraction (**B**), during the Valsalva maneuver (**C**), and during evacuation (**D**). Note the anterior rectocele during evacuation.

The X-rays are taken while the patient sits on the seat, arms folded across the chest, in the following incidences: at rest, during contraction, during the Valsalva maneuver, during evacuation, and during post-evacuation (Figure 2). It is important to impress upon the patient the importance of remaining in position, inclining the thorax, during the X-ray exposures.

### Analysis of demographic, clinical, and defecography data

Patients were stratified, by age, into three groups: young adult (< 40 years of age), comprising 10 (25.64%) of the 39 patients; adult (40-60 years of age), comprising 18 (46.16%); and elderly (> 60 years of age), comprising 11 (28.20%).

All images were archived in the DICOM format and transferred to commercially available workstations: Leonardo (Siemens AG Medical Solutions; Munich, Germany) and OsiriX (Pixmeo SARL; Geneva, Switzerland). Cases in which the examinations did not follow the protocol were excluded, as were those in which the images were not archived correctly. All images were analyzed, case by case, by two expert physicians working independently. Disagreements were resolved by consensus.

The following measurements were considered:

- Anorectal angle: The anorectal angle is formed by a straight line passing through the axis of the anal canal and another that passes through the posterior wall of the rectum (Figure 3A). Under normal conditions, it is expected that the mean anorectal angle, at rest, is 95º, with a physiological variation of 65-100º^(4)^. With contraction, the angle should decrease and become more acute, whereas it should increase, becoming more obtuse, due to straightening of the rectum, during evacuation.
  **Figure 3:**
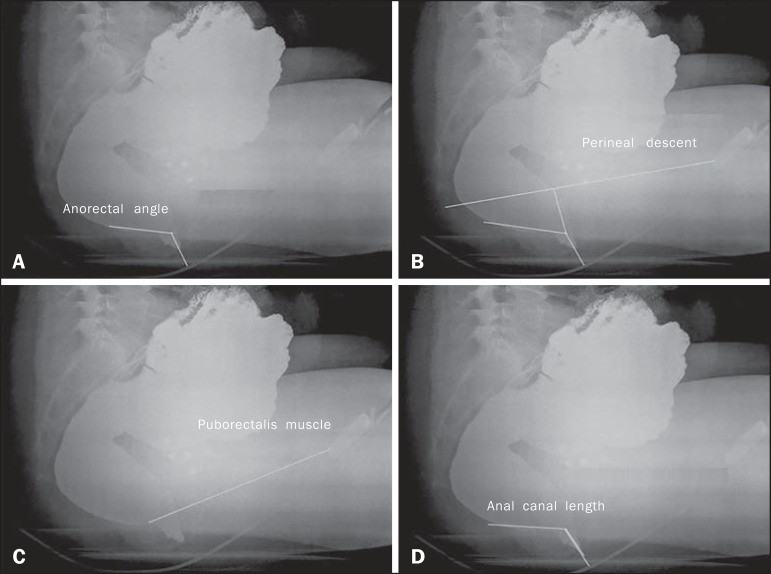
Anorectal angle (**A**), perineal descent (**B**), puborectalis muscle (**C**) and length of the anal canal (**D**) at rest.
- Perineal descent: First, a line is drawn from the pubic bone to the tip of the coccyx. The perineal descent is a line running the anorectal junction to a point at 90º on the pubococcygeal line (Figure 3B).
- Puborectalis muscle: This muscle is measured on a straight line that extends from the lower posterior portion of the pubic symphysis (landmark) to the point of greatest inflection in the posterior rectal wall (Figure 3C). The muscle typically relaxes, thus lengthening, during evacuation^(1)^.
- Anal canal length: The length of the anal canal is defined as the distance between the anal verge and the anorectal junction. The reference range is 2.5-4.0 cm (Figure 3D).
- Opening of the anal canal: The opening of the anal canal is measured in the anteroposterior direction, in centimeters. The reference value is 1.5 cm during evacuation^(5)^. It is important to take this measurement at rest, during contraction, and during evacuation, as well as to draw comparisons among the measurements, evaluating the capacity for contraction and relaxation. It is considered normal for there to be a difference of up to 3.5 cm between the measurement during evacuation and that obtained at rest^(5)^.
- Degree of rectal emptying: To determine the degree of rectal emptying, X-rays are obtained at rest and at one minute after evacuation. The degree of rectal emptying is calculated by subtracting the area measured in contrast-enhanced images before and after evacuation, using the following formula:

% of emptying = initial volume - final volume × 100

We consider > 80% emptying to be the reference value. Involuntary losses during the examination must be registered in order to characterize fecal incontinence.

### Statistical analysis

Continuous variables are expressed as mean ± standard deviation, and categorical variables are expressed as number and percentage, according to the situation. Comparisons among the age groups were made by analysis of variance with Bonferroni correction. The statistical analysis was performed with Stata^®^, version 12.0 (StataCorp LP; College Station, TX, USA) and Excel plug-in (Daniel's XL Toolbox, version 4.01; Daniel Kraus, Boston, MA, USA), the level of significance being set at < 0.05.

## RESULTS

Of the 39 patients evaluated, 37 (94.8%) were female and 2 (5.2%) were male. Patient ages ranged from 18 to 82 years, with a mean age of 52 ± 13 years. There were 10 patients (25.64%) in the young adult group (< 40 years of age), 18 (46.16%) in the adult group (40-60 years of age), and 11 (28.20%) in the elderly group (> 60 years of age).

All 39 examinations presented radiological changes. A total of 14 diagnoses were made, the most prevalent rectocele (Figure 2, shown during evacuation), which was observed in 30 patients (77%). A diagnosis of enterocele was established in 15 patients (38.40%), the same being true for contrast retention after evacuation. Rectal prolapse was identified in 13 patients (33.30%) and puborectalis muscle flaccidity was identified in an equal number of patients. Posterior rectocele was identified in 10 patients (25.60%). Other diagnoses were enterocele with compressive effect on the rectum, dyskinesia of the posterior wall of the rectum, puborectalis muscle hypertonia, fecal incontinence, vaginal prolapse, fecal incontinence, uterine prolapse, hemorrhoid, and Meckel's diverticulum. These findings are presented in Table 1.

**Table 1:** Principal diagnoses made by defecography in the population studied.

Diagnosis	N	%
Anterior rectocele	31	79.50
Enterocele	15	38.40
Contrast retention after evacuation	15	38.40
Rectal prolapse	13	33.30
Puborectalis muscle flaccidity	13	33.30
Posterior rectocele	10	25.60
Enterocele with compressive effect on the rectum	7	18.00
Dyskinesia of the posterior wall	4	10.25
Puborectalis muscle hypertonia	2	5.12
Fecal incontinence	2	5.12
Vaginal prolapse	1	2.56
Uterine prolapse	1	2.56
Hemorrhoid	1	2.56
Meckel’s diverticulum	1	2.,56

N, number of patients.

Quantitative variations among the groups are listed in Table 2. Emptying of the rectal ampulla was the only quantitative marker that showed significant variance among the age groups, being 54 ± 25 in the young adult group, 79 ± 12 in the adult group, and 74 ± 11 the elderly group (p < 0.01). Other lengths and angles did not vary significantly among the groups (p > 0.05).

**Table 2:** Quantitative analysis of defecography examinations, by age group.

		Age group			
	Total	Young adult	Adult	Elderly	*P*
Anorectal angle (º)					
At rest	102 ± 24	114 ± 28	94 ± 21	103 ± 19	0.09
Contraction	83 ± 21	91 ± 13	77 ± 25	86 ± 19	0.20
Valsalva maneuver	97 ± 24	100 ± 28	91 ± 22	105 ± 21	0.21
Evacuation	122 ± 22	119 ± 24	118 ± 22	133 ± 15	0.09
Perineal descent (cm)					
At rest (length)	4.0 ± 1.9	3.2 ± 1.1	4.2 ± 1.6	4.3 ± 3.3	0.41
Contraction (length)	3.0 ± 2.1	2.2 ± 1.9	3.3 ± 2.0	3.5 ± 2.7	0.37
Valsalva maneuver (length)	5.5 ± 2.4	5.8 ± 3.1	5.1 ± 1.9	5.7 ± 2.6	0.71
Evacuation (length)	7.7 ± 2.0	8.4 ± 1.1	7.4 ± 2.3	7.5 ± 2.3	0.45
Puborectalis muscle (cm)					
At rest	20 ± 5	20 ± 5	20.9 ± 3.7	19.8 ± 5.8	0.79
Contraction	19 ± 5	19 ± 6	20.4 ± 4.1	19.6 ± 5.8	0.77
Valsalva maneuver	21 ± 4	21 ± 3	20.9 ± 3.9	21.1 ± 4.1	0.99
Evacuation	20 ± 5	19 ± 6	21.1 ± 3.6	19.3 ± 5.4	0.56
Length of the anal canal (cm)					
At rest	4.6 ± 1.3	4.5 ± 1.2	4.7 ± 1.0	4.5 ± 1.8	0.89
Contraction	6.1 ± 1.6	6.1 ± 2.0	6.2 ± 1.6	5.8 ± 1.3	0.81
Valsalva maneuver	3.7 ± 1.6	3.2 ± 1.9	3.9 ± 1.6	3.8 ± 1.2	0.94
Evacuation	2.4 ± 1.2	2.6 ± 1.4	2.6 ± 1.4	2.5 ± 1.0	0.97
Opening of the anal canal (cm)					
At rest	0.9 ± 0.4	0.9 ± 0.3	1.0 ± 0.3	0.9 ± 0.5	0.72
Contraction	0.7 ± 0.3	0.7 ± 0.3	0.8 ± 0.2	0.7 ± 0.4	0.57
Valsalva maneuver	0.9 ± 0.5	0.8 ± 0.2	1.0 ± 0.5	1.1 ± 0.6	0.35
Evacuation	1.4 ± 0.4	1.4 ± 0.4	1.3 ± 0.4	1.3 ± 0.3	0.76
Emptying of the rectal ampulla (%)	70 ± 20	54 ± 25	79 ± 12	74 ± 11	0.001

## DISCUSSION

Imaging studies play an important role in the evaluation of diseases of the digestive system, as has been demonstrated in several recent studies conducted in Brazil^(7-15)^. In the present study, the majority of patients undergoing defecography were female and were between 18 and 82 years of age. Among the imaging findings, anterior rectocele was the most prevalent and emptying of the rectal ampulla was a significant quantitative marker.

It is important that each treatment facility standardize its technique for carrying out the examination, formulating protocols that include taking a full patient history, using well located and standardized markers, and taking accurate measurements in order to prevent diagnostic misinterpretations.

There is considerable variation in the values that are considered normal for each parameter, depending on the method employed by each examiner, and those values must always be interpreted in conjunction with the clinical data^(1)^. Of the 39 patients evaluated, 37 (94.8%) were female and 2 (5.2%) were male. The higher prevalence in women was expected and was previously reported by Sobrado et al.^(16)^.

One finding that called our attention was that 100% of the examinations presented alterations. That indicates, indirectly, that the methods employed correctly indicated the need for the examinations; that is, that the examinations were performed in individuals who were truly ill. Anterior rectocele was the diagnosis that was most prevalent in all of the patient subgroups, and we found that the changes of the pelvic floor were more related to multiple associated disorders, parity, and prior disease than to the age of the patient, as was also observed in the study conducted by Santos et al.^(17)^.

Other common findings were enterocele, contrast retention after evacuation, puborectalis muscle flaccidity, and posterior rectocele. All of these diagnoses have a direct effect on patient quality of life. Unlike the physical examination, defecography allows early diagnosis of multiple dysfunctions of the pelvic floor, which completely changes the surgical approach^(17)^.

In the present study, the quantitative analysis showed little variance among age groups, only emptying of the rectal ampulla being significant (p < 0.05).

We believe that quantification in medical imaging is of the utmost importance. In the present study, the data demonstrate that disturbances of evacuation do not depend on age group alone; such disturbances mainly depend on the degree of pelvic floor dysfunction, with or without the involvement of multiple compartments. Multiparous women with dystocia tend to show findings that are much worse than would be expected simply as a result of advancing age, as was also observed by Sobrado et al.^(16)^ and Santos et al.^(17)^.

## CONCLUSION

Although defecography is performed more often in women, both genders can benefit from the test. Defecography can be performed in order to detect complex disorders such as uterine and rectal prolapse, as well as to detect basic clinical conditions such as rectocele or enterocele.
